# Genetic and expression variations of cell cycle pathway genes in brain tumor patients

**DOI:** 10.1042/BSR20190629

**Published:** 2020-05-14

**Authors:** Anum Zehra Naqvi, Ishrat Mahjabeen, Saima Ameen, Malik Waqar Ahmed, Asad Ullah Khan, Zertashia Akram, Mahmood Akhtar Kayani

**Affiliations:** Cancer Genetics and Epigenetics Lab, Department of Biosciences COMSATS University, Islamabad, Pakistan

**Keywords:** Brain tumor, CCND1, Expression analysis, Polymorphism, RB1

## Abstract

The present study was designed to determine the association between the genetic polymorphisms/expression variations of *RB1* and *CCND1* genes and brain tumor risk. For this purpose, 250 blood samples of brain tumor patients along with 250 controls (cohort I) and 96 brain tumor tissues (cohort II) with adjacent control section were collected. Mutation analysis of *RB1* (rs137853294, rs121913300) and *CCND1* (rs614367, rs498136) genes was performed using ARMS-PCR followed by sequencing, and expression analysis was performed using real-time PCR and immunohistochemistry. The results showed homozygous mutant genotype of *RB1* gene polymorphism, rs121913300 (*P*=0.003) and *CCND1* gene polymorphism rs614367 (*P*=0.01) were associated significantly with brain tumor risk. Moreover, significant down-regulation of *RB1* (*P*=0.005) and up-regulation of *CCND1* (*P*=0.0001) gene was observed in brain tumor sections vs controls. Spearman correlation showed significant negative correlation between *RB1* vs proliferation marker, *Ki-67* (r = −0.291*, *P*<0.05) in brain tumors. Expression levels of selected genes were also assessed at protein level using immunohistochemical analysis (IHC) and signification down-regulation of *RB1* (*P*=0.0001) and up-regulation of *CCND1* (*P*=0.0001) was observed in brain tumor compared with control sections. In conclusion, it is suggested that polymorphisms/expression variations of *RB1* and *CCND1* genes may be associated with increased risk of brain tumor.

## Introduction

Brain cancer develops in brain or in spinal cord and is categorized into different types on the basis of their location and origin such as glioma, meningioma, pituitary adenomas, medulloblastoma and schwannoma [1]. Among these types, glioma also referred to as tumors of glial cells, are the most common type of primary brain tumors [2]. Gliomas are further classified into various categories as per their morphological appearance which includes astrocytomas, oligodendrogliomas, ependymomas and glioblastomas. According to World Health Organization (WHO) 2007 classification, gliomas are classified into four categories namely grades I, II, III and IV which are decided on the basis of severity [3,4]. Regardless of advancements in therapeutical procedures like radiotherapy, chemotherapy and surgical removal, the prognosis and survival of patients (especially glioblastoma) is still lacking [5,6]. Therefore, it is significant to illuminate the exact molecular cause of glioma/brain tumor, which may possibly support in the diagnosis and prognosis of brain tumor patients. Radiation exposure to head and inherited syndromes (which are rare) are among the established risk factors of brain tumorigenesis [7]. The exact molecular mechanism of these risk factors to increase the risk of tumor development in brain is still unknown. Nevertheless, it has been observed that radiation disturbs the G_1_/S and S phases of the cell cycle pathway and ultimately resulted in increased proliferation and tumorigenesis.

G_1_/S transition phase defines the fate of the cell based on the proliferation or apoptotic signals. Genes which are involved in the transition of G_1_/S phase transition are cyclin-dependent kinases, cyclins and retinoblastoma 1 gene [8–10]. *RB1* gene confines the cell in gap phase in absence of proliferation signals. Activated pRB protein binds with E2F transcription factor and halt transcription mechanism. RB1/E2F complex suppresses the transcription in quiescent cells [11]. Any polymorphism or expression variation in *RB1* gene may disrupt the transition of cell cycle phases. *RB1* gene is found to be mutated in many cancers including brain tumor [12]. Selected polymorphisms of *RB1* gene rs137853294 and rs121913300 are exonic single nucleotide nucleotides (SNPs). These SNPs result in aberrant mRNA structure and protein of *RB1* gene in breast cancer patients [13].

Another important cell cycle pathway gene is *CCND1*, which forms a complex with CDK4 and initiates the transition of gap phase into synthesis phase. CCND/CDK complex inactivates pRb protein through phosphorylation resulting E2F transcription factor starts progression of cell from G-phase to S-phase [14]. Any mutation in proto-oncogene *CCND1* may lead to carcinogenesis through abnormal cell proliferation of cell. Its expression also varies most of the cancers including brain tumor [15]. *CCND1* polymorphisms rs614367 and rs498136 are intergenic SNPs which affects the regulation and expression of *CCND1* gene in breast cancer and malignant melanoma, respectively [16,17].

A number of studies have been published for expression analysis of *RB1* and *CCND1* in different cancers including brain cancer. However, till now, no study has been reported to screen out the hotspot polymorphisms of *RB1* and *CCND1* genes along with expression variations of respective genes in brain tumor and different subtypes of brain tumor. Present study was designed to find out whether the polymorphisms or expressional variation in the *RB1* and *CCND1* genes can modify the risk for brain tumor, and if the effects of these polymorphisms differ in different pathological parameters of brain tumor patients.

## Materials and methods

### Specimen collection

Ethical board of both COMSATS Institute of Information Technology and collaborating hospital approved the proposal of present study. Two study cohorts of brain tumors were patients enrolled in present study. Study cohort I consisted of 250 blood samples of brain tumor patients and 250 age and sex matched controls. Selection criteria for patients included in cohort 1 comprised confirmed histological diagnosis of brain tumor, no preoperative therapy and availability of complete follow-up data. However, no restrictions related to histological subtypes of primary brain tumors were applied. Different subtypes such as anaplastic astrocytoma, anaplastic oligodendroglioma, anaplastic meningioma, meningioma, diffuse astrocytoma, choroid glioma, oligodendroglioma, eppendoma, atypical meningioma, GBM, diffuse medine glioma and pituitary adenomas were included in the present study. Inclusion criterion for controls included absence of prior history of cancer or precancerous lesions. Patients and controls suffering from any other familial disease (diabetes, blood pressure and cardiovascular impairment) were excluded from the present study. Cohort I was used for screening of hotspot polymorphisms of *RB1* and *CCND1* genes in brain tumor patients. Study cohort II consisted of 96 brain tumor tissues along with adjacent uninvolved healthy area used as controls. Samples of tumor core, the invasive edge of tumor and microscopically healthy mucosa (control) were obtained from each surgical section and stored in RNA at −80°C. Presence of tumor cells in the collected tissues was rectified by examination of frozen sections following Hematoxylin and Eosin stain (HE stain) by a consultant pathologist. Whereas, samples of control were obtained from macroscopically confirmed (by a pathologist) uninvolved healthy area more than 2 cm away from the tumor. Cohort II was used for the expression analysis of *RB1* and *CCND1* gene using the quantitative real-time PCR technique. Both brain tumor cohorts were collected after taking the consent from patients from Department of Neurosurgery, Pakistan Institute of Medical and Health Sciences (PIMS) Hospital in during 2015–2017. After obtaining informed consent, all individuals were personally interviewed using the specifically designed questionnaire. Information on age, gender, ethnic group and detailed exposure data on smoking was recorded.

### RNA and DNA extraction

Blood samples of cohort I were collected in ETDA vacutainers. DNA was extracted from blood samples through phenol-chloroform method [18]. DNA samples were stored in TE buffer at 4°C for further mutation analysis. In case of cohort II, tumor samples were collected in 15-ml Eppendorf tube containing RNA later solution. RNA was extracted from fresh tissue samples of both tumor and control through TRIzol method [19]. Extracted RNA was stored in DEPC at −20°C for expression analysis of selected genes. Extracted RNA and DNA were visualized and confirmed by performing 1% agarose gel electrophoresis.

### Polymorphism screening and sequencing

Polymorphisms for *RB1* and *CCND1* gene were selected from dbSNP database. The SNP selection criteria were based on minor allele frequency (MAF) > 0.05. The selected SNPs included rs137853294, rs121913300 for *RB1*, rs614367 and rs498136 for *CCND1*.

Genotyping was performed by the amplification-refractory mutation system polymerase chain reaction (ARMS-PCR). Primers for PCR amplification were designed using WASP (web-based allele specific primer designing tool) and purchased from Macrogen (Korea). Primers specific for each polymorphism are given in Table 1. PCR was performed in a reaction volume of 10 μl containing 50–100 ng genomic DNA, 100 μM of each primers and Solis BioDyne Master Mix. The thermal cycling protocol used was: 94°C for 5 min and 35 cycles at 94°C for 30 s, optimized annealing temperature for 45 s, 72°C for 1 min and final extension for 10 min. The PCR products were visualized on a 2% agarose gel electrophoresis (100 V, 300 A for 45 min). Presence or absence of expected product size bands, specific for wild or mutant primers, were evaluated using UV transilluminator (Gel Doc Bio-Rad, U.S.A.). β*-Actin* was used as internal control in each reaction. PCR products with homozygous wild, homozygous mutant and heterozygous mutant genotypes were further confirmed by DNA sequence analysis

**Table 1:** Primer sequence of *RB1* and *CCND1* genes

Primer name	Primer sequence	Product size
**Primers specific for real-time PCR**		
CCND1 F	CCTCGGTGTCCTACTTCAAATG (sense)	100 bp
CCND1 R	CACTTCTGTTCCTCGCAGAC (antisense)	
RB1 F	GCAGTATGCTTCCACCAG (sense)	108 bp
RB1 R	TAGATGTTCCCTCCAGGAAT (antisense)	
**Primers specific for ARMS-PCR**		
**RB1 first SNP**		
RB1 rs137853294 FI (T allele)	CAGTGTATCGGCTAGCCTATCGCT	188 bp
RB1 rs137853294 RI (C allele)	AAGCGTTCACAAAGTGTATTTAGACG	257 bp
RB1 rs137853294 FO (5′–3′)	TGCATTTCTTCATCTGTATCCCTTGT	394 bp
RB1 rs137853294 RO (5′–3′)	GTAGGGAGGAGAGAAGGTGAAGTGC	
**RB1 second SNP**		
RB1 rs121913300 FI (T allele)	AAGGTTGAAAATCTTTCTACAT	150 bp
RB1 rs121913300 RI (C allele)	AGATAAATTTCTTCGTAGCG	214 bp
RB1 rs121913300 FO (5′–3′)	CAATTTTCTGTACCTCACTTT	322 bp
RB1 rs121913300 RO (5′–3′)	CTAAAGCAAATCAATCAAATAT	
**CCND1 first SNP**		
CCND1 rs614367 FI (T allele)	TTGGCTTCTCTGCAACGCT	214 bp
CCND1 rs614367 RI (C allele)	GGGGCCTAAAGAGATGTAATTCG	293 bp
CCND1 rs614367 FO (5′–3′)	TTACATAGAAGGGGGTGAGCCA	465 bp
CCND1 rs614367 RO (5′–3′)	TGTTCTGTCCATTGTCCAGCAT	
**CCND1 second SNP**		
CCND1 rs498136 FI (T allele):	CATTATTGTGCTTCTCAACGGT	153 bp
CCND1 rs498136 RI (G allele)	AAACTCTTAAGTTTCACTGTTTGGTC	103 bp
CCND1 rs498136 FO (5′–3′)	CTGCCAGAGGACTTAGAAAGTG	208 bp
CCND1 rs498136 RO (5′–3′)	TAGACATGGAAAATATTCACGACA	

### Expression analysis

Expression variation of *RB1, CCND1* and *Ki-67* genes was analyzed by performing quantitative real-time PCR technique. cDNA was synthesized and confirmed by amplification of β-actin which was taken as endogenous control. Primers were designed for β-actin, *RB1, CCND1* and *Ki-67* genes by IDT software for real-time PCR to estimate the relative expression of these genes in control and tumor samples.

Each qPCR was performed in a 20-μl reaction mixture containing ∼1 μl of product from RT reaction, 10 μl of 2× SYBR Green, 1 μl of each primer and 7 μl of RNase-free water. qPCR was performed using Real-Time PCR system (Applied Biosystems Step One Plus) under standard conditions. The relative mRNA levels of *RB1, CCND1, Ki-67* and β-*actin* were computed using the 2^−ΔΔ*C*_t_^ analysis method [19]. Melt curve analysis of selected genes was also performed to detect the heterogeneity in brain tumor cohort and specificity or qPCR [20].

### Immunohistochemistry

Immunohistochemical analysis (IHC) was performed using the DAB kit (Sigma). Paraffin-embedded sections of brain tumor sections and adjacent uninvolved control sections were used for the present study after deparaffinization and rehydration steps using xylene and ethanol respectively, as described previously [21]. Sections were incubated for 1 h with mouse anti-RB1 (Novus Biologicals, at a dilution of 1:1000), mouse anti-CCND1 (Novus Biologicals, at a dilution of 1:1000) and mouse anti-Ki-67 (Novus Biologicals, at a dilution of 1:1000). Species-specific secondary antibody (Novus Biologicals) was also used for each selected gene. Additionally, negative controls were prepared using the same procedure except that the primary antibodies were replaced with PBS. Negative controls were used for assessment of specificity of this current assay [21].

The relative intensities of the completed immunohistochemical reactions were also evaluated using light microscopy by three independently trained observers, unaware of the clinical data. Tumor cells were counted randomly in ten high power fields for measurement of immunoreactivity. The following formula was used for the evaluation of immunoreactivity:

$$\text{Immunoreactive score}\;\frac{1}{4}\;\text{intensity score }\times \text{ propotion score}$$

### Structure prediction

Centroid secondary structure of mRNA of wild-type *RB1* transcript and its selected exonic SNPS were designed by dot block model using Veinna RNA Web Services software. Furthermore, protein structure of normal pRB and mutated pRB were predicted using SWISS-MODEL software.

### Statistical analysis

Statistical analysis was performed using MedCalc statistical software and GraphPad Prism (5.03) software. Comparison between the expected and actual genotypes was done using Hardy–Weinberg equilibrium test. Chi-square tests was applied to estimate the difference in genotypes and allele frequencies between patients and control individuals. Odds ratio (ORs) and 95% confidence intervals (CIs) were assessed through logistic regression. This predictive analysis was performed with respect to demographic features, smoking status, family history, tumor types and grading. SNPs analysis and their multiplicative interactions were also calculated by three genetic models (additive, dominant and recessive) and logistic regression model, respectively. *P*-value <0.05 represents the statistically significant difference between patients and controls. Genotyped data of SNPs were used to design their haplotypes using Haploview 4.2 software. Linkage disequilibrium (LD) between all selected SNPs was determined through expectation maximization (EM) algorithm.

For cohort II, Chi-square tests and one-way analysis of variance was used to assess the association of *RB1* and *CCND1* gene expression with clinical and histopathological parameters. Spearman correlation coefficient was used to assess correlations among the gene expression and clinical and histopathological parameters.

## Results

### Retrospective cohort study

In study cohort I, 250 brain tumor patients and 250 control individuals were examined for four selected SNPs including rs137853294, rs121913300 (*RB1*), rs614367 and rs498136 (*CCND1*). In study cohort II, 96 brain tumor tissues and 96 healthy tissues were analyzed for the expression variation of *RB1* and *CCND1* genes. Demographic features of the study cohort are given in Table 2.

**Table 2:** Demographic parameters of brain tumor cases and controls

Variables	Cases (*n*=250)	Controls (*n*=250)	Tumor tissues (*n*=96)
**Age (years)**			
Median (range)	42 (8–88)	47 (9–88)	45 (21–71)
**Age**			
≤30	62 (24.8%)	49 (19.6%)	12 (12.5%)
>30	188 (75.2%)	201 (80.4%)	84 (87.5%)
**Gender**			
Males	165 (66%)	172 (68.8%)	59 (61.5%)
Females	85 (34%)	78 (31.2%)	37 (38.5%)
**Smoking**			
Smokers	83 (33.2%)	120 (48%)	44 (46%)
Non-smokers	167 (66.8%)	130 (52%)	52 (54%)
**Family history**			
Positive	26 (10.4%)	-	13 (14%)
Negative	224 (89.6%)	-	83 (86%)
**Brain tumor types**			
**Glioma** Diffuse astrocytoma (II) Anaplastic astrocytoma (III) Choroid glioma (II) Oligodandroglioma (II) Anaplastic oligodandroglioma (III) GBM (IV) Diffuse medine glioma (IV) Eppendoma (II)	170 (68%) 35 64 25 06 04 27 06 03	-	68 (70.8%) 07 19 12 05 03 11 04 07
**Meningioma** Meningioma (I) Atypical meningioma (II) Anaplastic meningioma (III)	64 (25.6%) 21 30 13	-	21 (21.9%) 09 08 04
**Pituitary adenoma** Grade II Grade III	16 (6.4%) 09 07	-	07 (7.3%) 04 03
**Tumor grade**			
Grade I	21		09
Grade II	108		43
Grade III	88		29
Grade IV	33		15
**Ionizing radiation**			
Exposed	24 (9.6%)	-	16 (16.7%)
Unexposed	226 (90.4%)	-	80 (83.3%)

### Genotype and allele frequencies of *RB1* and *CCND1* SNPs

Genotype and allele frequencies of RB1 polymorphisms are mentioned in Table 3. *RB1* polymorphism rs137853294 showed significantly higher frequency of heterozygous mutant genotype (OR, 1.49; 95% CI, 1.02–2.16; *P*=0.04) in patients as compared with controls. Mutant allele (T) frequency of rs137853294 was significantly higher (OR, 1.50; 95% CI, 1.11–2.01; *P*=0.007) in tumor patients as compared with control individuals. In case of rs121913300 *RB1* polymorphism, ∼20.48-fold increased risk of brain tumor was associated with homozygous mutant genotype (OR, 20.48; 95% CI, 2.72–154.21; *P*=0.003) in tumor patients. Moreover, significantly higher frequency of heterozygous mutant genotype (OR, 1.68; 95% CI, 1.05–2.66; *P*=0.03) and mutant T allele (OR, 1.56; 95% CI, 1.22–2.01; *P*=0.0005) was observed in sampled tumor patients as compared with controls.

**Table 3:** Genotypes and allele frequency of selected SNPs of *RB1* and *CCND1* in brain tumor patients

Genotype/Allele	Cases, *n* (%)	Controls, *n* (%)	OR (95% CI)	*P*-value
***RB1 gene***				
**rs137853294**				
CC	135 (54%)	164 (65.6%)	1	1
CT	95 (38%)	73 (29.2%)	1.49 (1.02–2.16)	0.04*
TT	20 (8%)	13 (5.2%)	1.60 (0.77–3.26)	0.21
C allele frequency	365 (73%)	401 (80.2%)	1	1
T allele frequency	135 (27%)	99 (19.8%)	1.50 (1.11–2.01)	0.007^†^
**rs121913300**				
CC	17 (6.8%)	54 (21.6%)	1	1
CT	214 (85.6%)	195 (78%)	1.68 (1.05–2.66)	0.03*
TT	19 (7.6%)	1 (0.4%)	20.48 (2.72–154.21)	0.003^†^
C allele frequency	248 (49.6%)	303 (60.6%)	1	1
T allele frequency	252 (50.4%)	197 (39.4%)	1.56 (1.22–2.01)	0.0005^‡^
***CCND1 gene***				
**rs614367**				
CC	119 (47.6%)	154 (61.6%)	1	1
CT	117 (46.8%)	93 (37.2%)	1.48 (1.04–2.12)	0.03*
TT	14 (5.6%)	3 (1.2%)	4.88 (1.39–17.21)	0.01*
C allele frequency	355 (71%)	401 (80.2%)	1	1
T allele frequency	145 (29%)	99 (19.8%)	1.65 (1.23–2.22)	0.0007^‡^
**rs498136**				
GG	85 (34%)	104 (41.6%)	1	1
GT	157 (62.8%)	145 (58%)	1.22 (0.85–1.75)	0.27
TT	8 (3.2%)	1 (0.4%)	8.23 (1.02–66.31)	0.047*
G allele frequency	327 (65.4%)	353 (70.6%)	1	1
T allele frequency	173 (34.6%)	147 (29.4%)	1.27 (0.97–1.66)	0.078

Abbreviation: *n*, number of samples. Level of significance: *P*-value <0.05*, <0.01^†^, <0.001^‡^.

*CCND1* polymorphism’s genotype and allele frequencies are given in Table 3. Genotype frequency of *CCND1* polymorphism rs614367 showed ∼4.88-fold increased risk of brain tumor with homozygous mutant genotype (OR, 4.88; 95% CI, 1.39–17.21; *P*=0.01) in sampled tumor patients. Furthermore, higher frequency of heterozygous mutant genotype (OR, 1.48; 95% CI, 1.04–2.12, *P*=0.03) and mutant T allele (OR, 1.65; 95% CI, 1.23–2.22; *P*=0.0007) was observed in tumor patients as compared with control individuals. In case of rs498136 polymorphism of *CCND1*, ∼8.23-fold increased risk of brain tumor was observed associated with homozygous mutant genotype (OR, 8.23; 95% CI, 1.02–66.31; *P*=0.047) in brain tumor patients compared with controls.

### Logistic regression models analysis

Three logistic regression models (dominant, recessive, additives) were used for further analyses of genotype frequencies of all SNPs as shown in Table 4. For *RB1* SNP rs137853294, dominant (OR, 1.62; 95% CI, 1.13–2.33; *P*=0.008) and additive genetic model (OR, 1.50; 95% CI, 1.11–2.01; *P*=0.007) showed significant association with increased risk of brain tumor. Similarly, rs121913300 SNP showed the significant association of brain tumor risk with all three genetic models, i.e. dominant (*P*<0.0001), recessive (*P*=0.003) and additives (*P*=0.0005). Moreover, *CCND1* polymorphism rs614367 showed significant association of all three genetic models, i.e. dominant (*P*=0.002), recessive (*P*=0.01) and additive (*P*=0.0007) with increased risk of brain tumor. rs498136 polymorphism also showed significant association of recessive genetic model (OR, 8.23; 95% CI, 1.02–66.31; *P*=0.047) with increased risk of brain tumor.

**Table 4:** Association of SNPs of *RB1* and *CCND1* gene with brain tumor risk based on genetic models

Genotype/Allele	Model	OR (95% CI)	*P*-value
***RB1 gene***			
**rs137853294**			
CC vs CT + TT	Dominant	1.62 (1.13–2.33)	0.008^†^
TT vs CC + CT	Recessive	1.58 (0.77–3.26)	0.21
T vs C	Additive	1.50 (1.11–2.01)	0.007^†^
**rs121913300**			
CC vs CT + TT	Dominant	3.78 (2.12–6.73)	<0.0001^****^
TT vs CC + CT	Recessive	20.48 (2.72–154.21)	0.003^†^
T vs C	Additive	1.56 (1.22–2.01)	0.0005^‡^
***CCND1 gene***			
**rs614367**			
CC vs CT + TT	Dominant	1.77 (1.24–2.52)	0.002^†^
TT vs CC + CT	Recessive	4.88 (1.39–17.21)	0.01*
T vs C	Additive	1.65 (1.23–2.22)	0.0007^‡^
**rs498136**			
GG vs GT + TT	Dominant	1.38 (0.96–1.99)	0.08
TT vs GG +GT	Recessive	8.23 (1.02–66.31)	0.047*
T vs G	Additive	1.27 (0.97–1.66)	0.08

### Association of *RB1* and *CCND1* SNPs with different parameters

Genotypes of all four selected SNPs of cell cycle genes were associated with demographic features, risk factors and histopathological subtypes by applying logistic regression model as shown in Table 5. Results showed that rs137853294 polymorphism (OR, 2.272; 95% CI, 0.088–0.084; *P*=0.024) and rs121913300 polymorphism of *RB1* (OR, 0.264; 95% CI, 0.082–0.0851; *P*=0.026) showed positive association with tumor grades. For *CCND1* gene, rs614367 and rs498136 also showed positive association with tumor grades (OR, 4.197; 95% CI, 0.054–0.771; *P*=0.014 and OR, 2.137; 95% CI, 0.258–5.007; *P*=0.03), as shown in Table 5.

**Table 5:** Association of selected SNPs of *RB1* and *CCND1* genes with different parameters

SNPs vs parameters	B	Std. error	Wald	Sig.	OR	95% CI
rs137853294 vs						
Gender	−0.636	0.533	1.428	0.232	0.529	0.186–1.503
Age	0.399	0.558	0.512	0.474	1.491	0.499–4.451
Smoking	0.33	0.614	0.289	0.591	0.719	0.216–2.394
Family history	−0.877	0.783	1.255	0.263	0.416	0.090–1.929
IR	−0.07	1.281	0.003	0.956	0.932	0.076–11.490
**Grade**	**3.3**	**0.577**	**5.077**	**0.024**	**2.272**	**0.088–0.844**
Types	−0.536	0.603	0.790	0.374	0.585	0.180–1.907
rs121913300 vs						
Gender	−0.127	0.524	0.059	0.809	0.881	0.316–2.459
Age	0.332	0.617	0.289	0.591	1.393	0.416–4.668
Smoking	1.958	0.518	3.418	0.064	1.384	0.139–1.059
Family history	0.247	0.509	0.235	0.628	1.28	0.472–3.470
IR	0.061	0.823	0.005	0.941	1.062	0.212–5.327
**Grade**	**4.333**	**0.598**	**4.976**	**0.026**	**3.264**	**0.082–0.851**
Types	-0.405	0.596	0.462	0.497	0.667	0.208–2.144
rs614367 vs						
Gender	−0.017	0.616	0.001	0.978	0.983	0.294–3.285
Age	0.168	0.659	0.065	0.798	1.183	0.325–4.304
Smoking	1.589	0.678	5.496	0.019	0.204	0.054–0.771
Family History	0.572	1.139	0.253	0.615	1.772	0.190–16.512
IR	0.334	1.159	0.083	0.773	1.397	0.144–13.537
**Grade**	**5.625**	**0.659**	**6.084**	**0.014**	**4.197**	**0.054–0.716**
Types	−0.249	0.685	0.133	0.716	0.779	0.204–2.983
rs498136 vs						
Gender	0.56	0.864	0.42	0.517	1.75	0.322–9.517
Age	−1.052	0.777	1.834	0.176	0.349	0.076–1.601
Smoking	−0.586	0.759	0.597	0.44	0.556	0.126–2.462
Family history	0.124	1.162	0.011	0.915	1.132	0.116–11.049
IR	−0.822	1.201	0.468	0.494	0.44	0.042–4.625
**Grade**	**2.128**	**0.756**	**0.029**	**0.035**	**2.137**	**0.258–5.007**
Types	0.788	1.117	0.498	0.480	2.200	0.247–19.633

Bold values are statistically significant. Level of significance: *P*-value <0.05.

### Haplotype analysis of *RB1* and *CCND1* SNPs

LD was estimated between all selected SNPs of *RB1* and *CCND1* genes. Haplotypes of these SNPs were also examined to determine their association with tumor risk. Thirteen common haplotypes were generated from these selected SNP, among brain tumor patients and controls as shown in Table 6. Haplotypes which significantly increased the risk of brain tumor included CCTG (*P*=0.002), CCTT (*P*=3.57e-006), CTCG (*P*=5.41e-010), TCCT (*P*=0.008), TCTG (*P*=0.006), TCTT (*P*=0.0006), TTTT (*P*=0.01) and CTCT (*P*=2.16e-006). Whereas, haplotypes which significantly reduced the risk of brain tumor included CCCG (*P*=6.21e-012), CCCT (*P*=1.09e-009), CTTG (*P*=0.0005) and TTTG (*P*=0.003).

**Table 6:** Haplotypes of cell cycle genes (*RB1* and *CCND1*) in brain tumor patients

*RB1 haplotypes (SNPs)*		*CCND1 haplotypes (SNPs)*		*Frequency*		χ²	*P*-value	OR	95% CI
rs137853294	rs121913300	rs614367	rs498136	Cases	Controls				
C	C	C	G*	0.107	0.279	47.336	6.21e-012	0.31	0.219–0.437
C	C	C	T*	0.091	0.234	37.222	1.09e-009	0.329	0.228–0.476
C	C	T	G*	0.054	0.018	9.456	0.002113	3.127	1.458–6.707
C	C	T	T*	0.074	0.014	21.51	3.57e-006	5.717	2.506–13.040
C	T	C	G*	0.295	0.134	38.583	5.41e-010	2.711	1.966–3.739
C	T	T	G*	0.06	0.124	12.078	0.000513	0.453	0.288–0.715
T	C	C	G*	0.035	0.018	2.709	0.099806	1.962	0.867–4.440
T	C	C	T*	0.058	0.025	6.845	0.008905	2.403	1.223–4.721
T	C	T	G*	0.021	0.003	7.507	0.006161	8.424	1.361–52.138
T	C	T	T*	0.056	0.016	11.582	0.00067	3.642	1.648–8.044
T	T	C	G*	0.081	0.109	2.213	0.136876	0.724	0.473–1.110
T	T	T	G*	0.001	0.022	8.746	0.003113	0.053	0.007–0.376
T	T	T	T*	0.017	0.001	6.109	0.013468	11.843	1.815–77.292

Abbreviation: χ², chi-square. *P*-value <0.05 was statistically significant.

**Figure 1:**
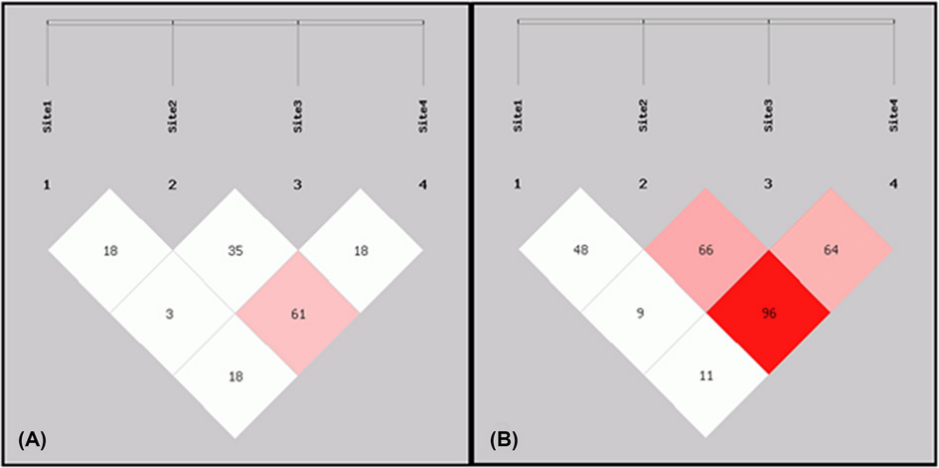
LD plot for *RB1* and *CCND1* polymorphism (**A**) Control sample; (**B**) tumor patient samples. Site1 for rs137853294; Site2 for rs121913300; Site3 for rs614367; Site4; rs498136; darker region shows higher r^2^-value.

Moreover, LD plot showed strong LD between exonic *RB1* SNP (rs121913300) and intergenic *CCND1* SNP (rs614367) in tumor patients. Highly strong LD was also observed between *CCND1* intergenic SNPs (rs614367 and rs498136) in brain tumor patients compared with controls as illustrated in Figure 1. Logistic regression analysis for SNP–SNP interactions among all four SNPs of *RB1* and *CCND1* genes showed positive significant correlation between s137853294 vs rs498136 (OR, 3.372; 95% CI, 0.520–21.872; *P*=0.02) and rs614367 vsrs498136 (OR, 3.083; 95% CI, 0.559–17.00; *P*=0.029) with increased risk of brain tumor as shown in Table 7.

**Table 7:** SNP–SNP interactions of all selected SNPs with brain tumor risk based on logistic regression model

SNP–SNP interactions	B	Std. error	Wald	Sig.	OR	95% CI
rs137853294 vs rs121913300	0.852	0.756	1.269	0.26	2.343	0.532–10.315
rs137853294 vs rs614367	1.509	1.153	1.715	0.19	0.221	0.023–2.116
rs137853294 vs rs498136	3.329	0.954	1.624	0.0203	3.372	0.520–21.872
rs121913300 vs rs614367	1.064	0.738	2.076	0.15	2.897	0.682–12.314
rs121913300 vs rs498136	0.078	1.14	0.005	0.945	1.081	0.116–10.106
rs614367 vs rs498136	3.126	0.871	1.67	0.0296	3.083	0.559–17.006

*P*-value<0.05 was statistically significant.

### mRNA secondary structure and protein structure prediction of *RB1* SNPs

**Figure 2:**
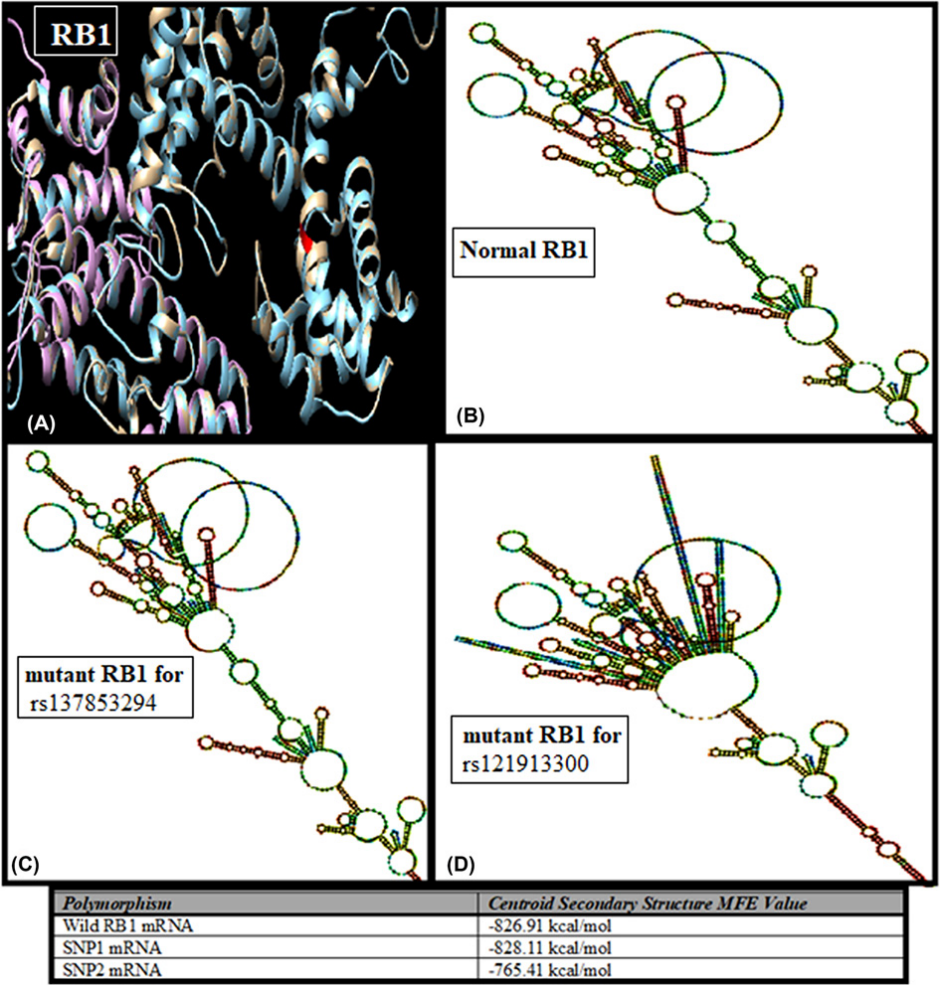
Protein and mRNA secondary structure prediction of *RB1* exonic SNPs (**A**) Super-imposed structure of normal and mutated *RB1* proteins (blue, wild; white, SNP1 mutated); purple, SNP2 truncated). (**B**) mRNA secondary structure of normal *RB1* gene. (**C**) mRNA secondary structure of SNP1 rs137853294 polymorphism. (**D**) mRNA secondary structure of SNP2 rs121913300 polymorphism.

Sequence of wild *RB1* gene and its two exonic SNPs were used to predict their secondary structure of mRNA using ViennaRNA Web Service. Prediction showed that there is no visual change in the structure of wild *RB1* mRNA and first missense mutated mRNA. While second mutated mRNA changed and shortened its structure as demonstrated in Figure 5. According to centroid secondary structure, minimum free energy (MFE) of wild mRNA and mutated mRNA were −826.91 and −828.11 kcal/mol, respectively. Moreover, truncated mRNA had −765.41 kcal/mol minimum free energy. Protein structure of wild *RB1* gene, SNP1 mutated *RB1* and SNP2 truncated *RB1* gene were predictive using SWISS-MODEL as illustrated in Figure 2.

### Expression analysis of *RB1* and *CCND1* gene at mRNA

**Figure 3:**
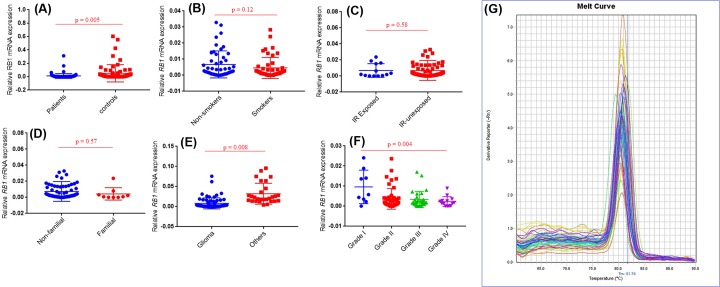
Expression profiling of *RB1* gene in study cohort mRNA expression of (**A**) *RB1* in brain tumor samples and normal control samples. (**B**) *RB1* in brain tumor samples with smoking status. (**C**) *RB1* in brain tumor samples with IR. (**D**) *RB1* in tumor samples with family history. (**E**) *RB1* in different types of brain tumor. (**F**) *RB1* in brain tumor samples with different grades. Among these grades, grade I included meningiomas (9), grade II included diffuse astrocytoma (7), choroid glioma (12), oligodandroglioma (5), eppendoma (7), atypical meningioma (8) and pituitary adenoma (4). Grade III inculded anaplastic astrocytoma (19), anaplastic oligodandroglioma (3), anaplastic meningioma (4) and pituitary adenoma (3). Grade IV included GBM (11) and diffuse medine glioma (4). (**G**) Melt curve analysis of *RB1* gene.

**Figure 4:**
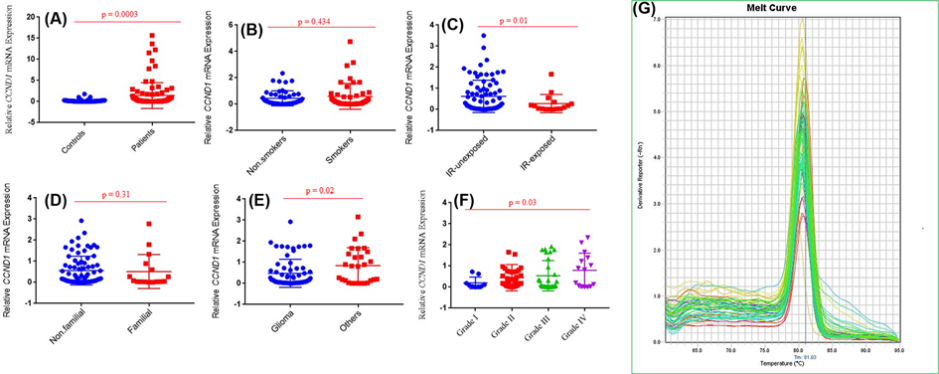
Expression profiling of *CCND1* gene in study cohort mRNA expression of (**A**) *CCND1* in brain tumor samples and normal control samples, (**B**) *CCND1* in brain tumor samples with smoking status, (**C**) *CCND1* in brain tumor samples with IR, (**D**) *CCND1* in tumor samples with family history, (**E**) *CCND1* in different types of brain tumor, (**F**) *CCND1* in brain tumor samples with different grades. Among these grades, grade I included meningiomas (9), grade II included diffuse astrocytoma (7), choroid glioma (12), oligodandroglioma (5), eppendoma (7), atypical meningioma (8) and pituitary adenoma (4). Grade III inculded anaplastic astrocytoma (19), anaplastic oligodandroglioma (3), anaplastic meningioma (4) and pituitary adenoma (3). Grade IV included GBM (11) and diffuse medine glioma (4). (**G**) melt curve analysis of *CCND1* gene.

*RB1* and *CCND1* mRNA levels were observed in study cohort II including 96 brain tumor tissues and adjacent uninvolved healthy section used as control. *RB1* expression has observed significantly lower in brain tumour (*P*=0.005) than in normal tissue samples. Statistical significant decrease in *RB1* mRNA level was observed in brain tumors with anatomical site of glioma (*P*=0.008) compared with other anatomical sites. Significant decreased mRNA level of *RB1* gene was also observed in grade IV (*P*=0.004) brain tumor as compared with other grades (Figure 3). In case of second selected gene *CCND1*, significantly higher mRNA level was observed in brain tumors section compared with adjacent control section (*P*=0.0003). Further analysis showed significantly increased *CCND1* mRNA level in grade IV (*P*=0.03) brain tumor patients compared with other grades (Figure 4).

**Figure 5:**
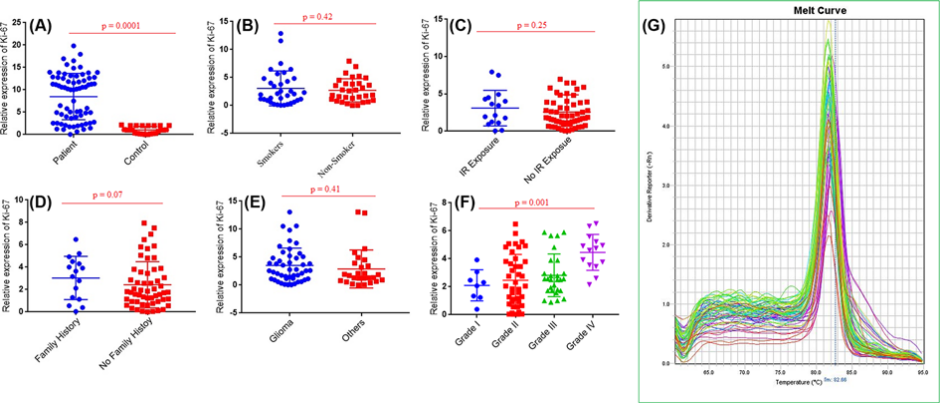
Expression profiling of Ki-67 gene in study cohort mRNA expression of (**A**) *Ki-67* in brain tumor samples and normal control samples, (**B**) *Ki-67* in brain tumor samples with smoking status (**C**) *Ki-67* in brain tumor samples with IR. (**D**) *Ki-67* in tumor samples with family history, (**E**) *Ki-67* in different types of brain tumor, (**F**) *Ki-67* in brain tumor samples with different grades. Among these grades, grade I included meningiomas (9), grade II included diffuse astrocytoma (7), choroid glioma (12), oligodandroglioma (5), eppendoma (7), atypical meningioma (8) and pituitary adenoma (4). Grade III inculded anaplastic astrocytoma (19), anaplastic oligodandroglioma (3), anaplastic meningioma (4) and pituitary adenoma (3). Grade IV included GBM (11) and diffuse medine glioma (4). (**G**) Melt curve analysis of *Ki-67* gene.

Expression level of proliferation marker *Ki-67* was also observed to find out whether the change in expression level of cell cycle gene affected the proliferation process in brain tumorigenesis. Significantly higher level of Ki-67 was observed in brain tumor (*P*=0.0001) compared with adjacent control section and this up-regulation was more pronounced in grade IV brain tumor tissue (*P*=0.001) compared with other grades (Figure 5).

### Expression analysis of *RB1* and *CCND1* gene at protein level

**Figure 6:**
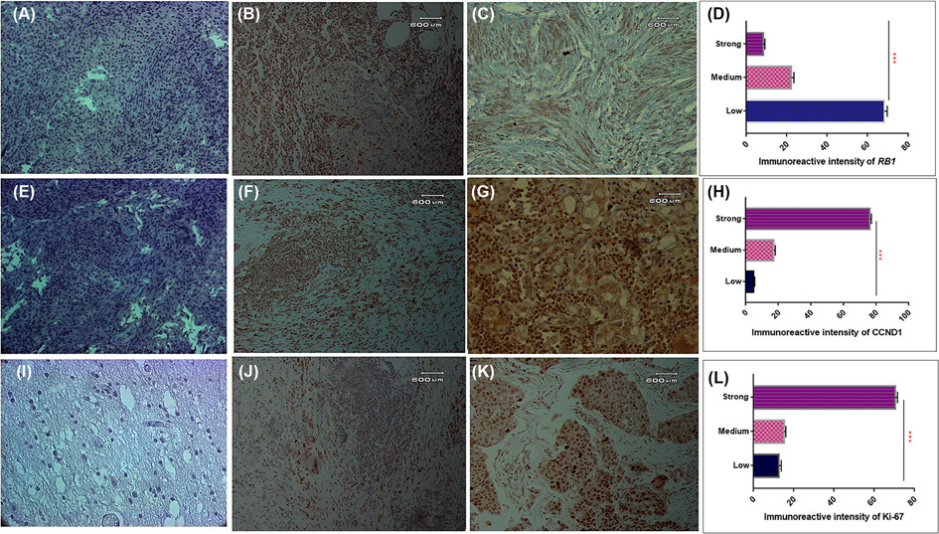
Immunohistochemical analysis of cell cycle pathway genes in study cohort Protein expression of *RB1* gene (**A**), negative control (**B**), adjacent control section (**C**) brain tumor. Immunoreactive intesity of *RB1* gene in brain tumor sections (**D**). Protein expression of *CCND1* gene (**E**), negative control (**F**), adjacent control section (**G**) brain tumor. Immunoreactive intesity of *CCND1* gene in brain tumor sections (**H**). Protein expression of *Ki-67* gene (**I**), negative control (**J**), adjacent control section (**K**) brain tumor. Immunoreactive intesity of *Ki-67* gene in brain tumor sections (**L**).

Expression level of *RB1, CCND1* and *Ki-67* was also observed in 96 brain tumor section and adjacent control section using IHC. Expression of *RB1* was observed lower in brain tumors compared with adjacent control sections as shown in Figure 6A,B,C. Measurement of immunoreactive intensity showed that weak immunoreactivity of *RB1* was observed significantly higher (*P*=0.001) in brain tumor sections as compared with moderate and strong immunoreactive intensity as shown in Figure 6D. Expression analysis of *CCND1* at protein level showed the increased expression of *CCND1* in brain tumor section compared with control section as shown in Figure 6E,F. Measurement of immunoreactive intensity also showed that strong immunoreactive intensity of *CCND1* was observed significantly higher (*P*=0.001) in brain tumor sections as compared with weak and moderate immunoreactive intensity as shown in Figure 6G.

Expression level of *Ki-67* was also observed at protein level using the IHC and expression level of *Ki-67* was observed to be higher in brain tumors section compared with adjacent control section as shown in Figure 6H,I. Measurement of immunoreactive intensity also showed that strong immunoreactive intensity of *Ki-67* was observed significantly higher (*P*=0.001) in brain tumor sections as compared with weak and moderate immunoreactive intensity as shown in Figure 6J.

### Correlation between *RB1, CCND1* and *Ki-67* expression and clinicopathological characteristics

Correlations were tested between *RB1, CCND1* and *Ki-67*, clinical and pathological features of brain tumor patients group II. The *RB1* mRNA level was negatively correlated with grades (r = −0.252**, *P*<0.01), however positive correlation was observed for *CCND1* mRNA level and grades (r = 0.222*, *P*<0.05) of brain tumor patients. In case of gene to gene correlation, a significant negative correlation was observed between *CCND1* vs *RB1* (r = −0.337**, *P*<0.001) and *RB1* vs *Ki-67* (r = −0.291*, *P*<0.05) in brain tumor patients as shown in Table 8.

**Table 8:** Spearman correlations among clinical features and cell cycle pathway genes expression of brain tumors^*^

mRNA	Gender	Type	Survival	Family	Grade	I.R	Area	RB1	CCND1	Ki-67
Age	0.039	−0.037	0.074	0.111	0.06	−0.247‡	−0.22	−0.33	0.126	0.124
Gender		0.02	0.416	0.067	−0.04	0.016	0.195†	0.009	0.104	0.33‡
Type			0.056	−0.058	0.146	−0.027	−0.016	−0.067	−0.132	0.07
Survival				−0.057	0.01	0.108	0.049	−0.150	−0.05	0.171
Family					−0.182^†^	−0.081	0.005	0.137	−0.016	−0.169
Grade						0.045	−0.117	−0.252†	0.222†	0.103
I.R							−0.051	−0.098	0.072	0.091
Area								0.045	0.047	−0.264†
RB1									−0.337‡	−0.291†
CCND1										0.135

Protein level	Gender	Type	Survival	Family	Grade	I.R	Area	RB1	CCND1	Ki-67
Age	0.039	−0.037	0.074	0.111	0.06	−0.247§	−0.22	−0.29	0.12	0.07
Gender		0.02	0.416	0.067	−0.04	0.016	0.195†	0.32	0.09	0.05
Type			0.056	−0.058	0.146	−0.027	−0.016	−0.12	−0.11	0.13
Survival				−0.057	0.01	0.108	0.049	−0.08	−0.07	0.08
Family					−0.182†	−0.081	0.005	0.06	0.02	0.14
Grade						0.045	−0.117	−0.394‡	0.19	0.54§
I.R							−0.051	−0.16	0.06	0.16
Area								0.08	0.12	0.21†
RB1									−0.54§	−0.29†
CCND1										0.43‡

^*^Spearman correlation coefficients. The expression levels of RB1, CCND1 and Ki-67 for the patient cohort were based on the relative mRNA level and protein level. The *P*-values were computed using one-way ANOVA and χ^2^-test.

† *P*<0.05.

‡ *P*<0.01.

§ *P*<0.001.

Correlations was also tested at protein level between selected genes and clinicopathological parameters of gastric cancer patients as shown in Table 8. A significant negative correlation was observed between *CCND1 vs RB1* (r = −0.54***, *P*<0.0001) and *RB1 vs Ki-67* (r = −0.29*, *P*<0.05) in brain tumor patients. However, a significant positive correlation was observed between *CCND1 vs Ki-67* (r = 0.43**, *P*<0.001) in brain tumor patients as shown in Table 8.

### ROC curve analysis

**Figure 7:**
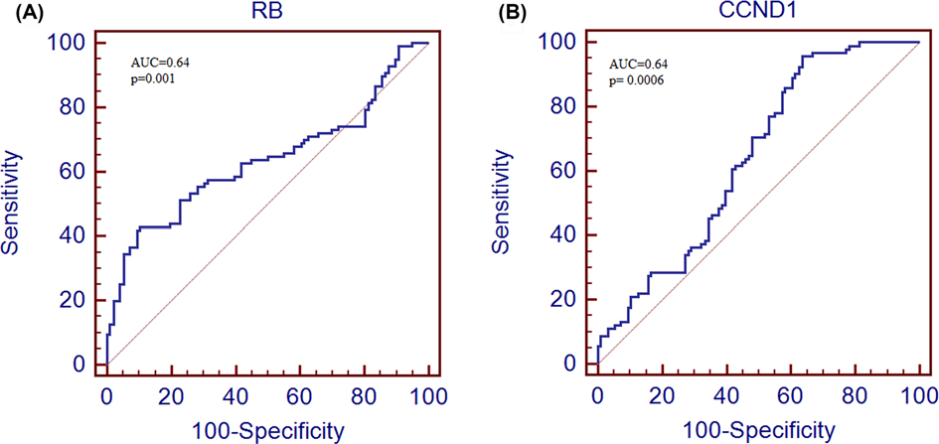
ROC curve analysis of cell cycle pathway genes in study cohort ROC curve anlysis of *RB1* gene (**A**) and *CCND1* (**B**) in brain tumor patients.

To assess the diagnostic value of both selected genes ROC curve analysis was performed. After the generation of ROC curve, area under the curve (AUC) and 95% CI was also calculated. The area under the curve for *RB1* gene was 63.5 (95% CI, 0.562–0.703; *P*<0.001) and for *CCND1* gene was 64.0 (95% CI, 0.567–0.709; *P*<0.0006) as shown in Figure 7.

## Discussion

The present study was designed to explore the correlation between the genetic polymorphism of *RB1* and *CCND1* genes to brain tumor risk in Pakistani population. The present study encompasses the genetic analysis of *RB1* exonic and *CCND1* intergenic polymorphisms in brain tumor patients to find their association with brain carcinogenesis. This has followed by structure prediction of exonic variants performed to assess the effect of these polymorphism on transcription level. Expression levels of *RB1* and *CCND1* were also analyzed in brain tumor patients. Furthermore, expression levels of cell cycle pathway genes were also correlated with proliferation marker, *Ki-67* to illuminate correlation between cell cycle pathway gene variation and proliferation process in brain tumorigenesis.

In present study we used two study cohorts, cohort I consisted of 250 blood samples of brain tumor patients and cohort II consisted of 96 brain tumors. Both cohorts of brain cancer contained higher number of glioma patients compared with other type of brain tumors such as meningioma and pituitary adenomas. Previous studies have also reported the increased incidence of glioma (77–81% of all primary malignant tumors of CNS) in the southeast, northwest, and midwest of U.S.A. [22], Asian population and Hispanic population [23] compared with meningioma and pituitary adenomas. Reasons binding these incidence differences can be variations in geographic regions, environmental factors, diet, occupational and personal exposures and lifestyle [22,24]. In addition, ethnic/race variations are likely to contribute to observed differences [25,26].

Retinoblastoma 1 gene has been found mutated or deregulated in many human cancers [27–35] and one subtype of brain tumor, glioma [36]. Present study examined the two exonic SNPs of *RB1* gene (rs137853294 and rs121913300) in brain tumor patients. Exonic or coding region SNPs mainly influence the transcription of the gene by altering the structure and function of transcribed protein [37]. Among SNPs, rs137853294 showed the significant association in heterozygous mutant genotype to brain tumor risk in current study. This SNP alters the protein structure of *RB1* by replacing amino acid Arginine to Tryptophan. As a result of this missense mutation, altered pRB protein is formed which disrupts the cell cycle checkpoints and contributes to tumorigenesis [13]. Furthermore, second exonic variant, rs121913300 was also observed significantly associated with brain tumor risk, indicated by genotypic frequency of both homozygous and heterozygous mutants in current study. This SNP stops the transcription of *RB1* gene by inducing the stop codon. Resulting truncated pRB protein is formed which causes the abnormality in cell cycle control [13]. These findings of both SNPs agree with many earlier studies which state that genetic mutations in *RB1* gene vary its expression and can contribute in retinoblastoma [13,27].

*Cyclin D1* gene, second important molecule of cell cycle pathway has also been found mutated in almost every human cancer by altering the normal cell cycle pathway. *CCND1* gene polymorphisms have been found in several cancers [38–47] including gliomas also [48]. However, till now no study has been published with respect to brain tumor and different subtypes of brain tumor such as glioma, meningioma and pituitary adenoma. In this study, two intergenic SNPs (rs614367 and rs498136) of *CCND1* were analyzed which can potentially influence the regulation and expression of *CCND1* gene. Any polymorphism in intergenic regions may disrupt the transcription of *CCND1* gene by altering its expression and may increase the susceptibility of diseases [49].

SNP rs613467 is present in the regulatory region of *CCND1* which regulates the transcription of *CCND1* gene. Alterations in that region can cause the abnormality in interpretation of regulating signals resultantly producing transcription abnormalities [50]. rs614367, was found significantly associated with increased risk of brain tumor in both homozygous and heterozygous mutant genotypes. Similar trend of rs614367 association has earlier been found in breast cancer patients in earlier studies [51,52]. SNP rs498136 is located in 5′ promotor region of *CCND1* at transcription binding site and resultantly alters the binding site of transcription factors for *CCND1* gene and aberrant regulation of the gene [53]. Second selected SNP of *CCND1* polymorphism rs498136, was also showed significant association in brain tumor patients in homozygous mutant genotype in present study. Findings of the present study are in line with the previous study suggested the strong association of rs498136 with malignant melanoma [16].

To test the hypothesis that SNPs in coding region/intragenic region result in variations at transcription level and change expression level of respective genes. Expression levels of *RB1* and *CCND1* gene was also measured in cohort II of brain tumor patients and controls using qPCR and immunohistochemistry. Significant down-regulation of *RB1* expression was observed in brain tumor patients when compared with adjacent controls, after qPCR and IHC. This down-regulation was observed as more pronounced in grade IV tumors compared with lower grade tumors. Expression pattern variations between the controls and patients may be due to genetic heterogeneity in *RB1* gene by different environmental factors and genetic and epigenetic changes [54,55].

Expression level of *RB1* gene was also correlated with different type of brain tumors and significant down-regulation of RB1 gene was observed in glioma compared with meningioma and pituitary adenomas. Deregulation of RB1 gene has been reported in different types of brain tumor such as glioma [56] and meningioma [57]. It has also been reported in previous study that down-regulation of *RB1* gene in glioma was associated with increased cell proliferation and decrease survival in more than one-third of patients [58]. One of basic reason of down-regulated expression of *RB1* gene in glioma compared with meningioma and pituitary adenoma, can be that number of gliomas are significantly higher in our study cohort compared with other type of brain tumors. Other reasons of this down-regulation are genetic variations [30,59], *RB1* promoter hypermethylation [31,60–63], variations in microRNA binding affinity [64,57] and this down-regulated/loss of expression ultimately resulted in increased invasion of tumor cells and more aggressive type of brain tumor in CNS carcinogenesis process [62].

In case of second selected molecule, *CCND1* gene, significant up-regulation was observed in brain tumor patients compared with controls using qPCR and immunohistochemistry. Additionally, this up-regulation was observed higher in aggressive brain tumors in present study. *CCND1* overexpressed and gene amplification is reported in many cancers including the meningioma and pituitary adenomas [65–68]. Mechanisms which are involved in over expression of *CCND1* gene by upstream factors, remain unknown. Genetic variations in exonic region [42] and intragenic/UTR region resulted in abnormal binding of transcription factors. [67] has found the relation between abnormal binding of octamer-binding transcription factor 4 (OCT4) due to polymorphism in UTR region of *CCND1* with the octamer motif (ATTTTGCAT) and promoted proliferation of cell cycle [66] and tumorigenesis. Additionally, expression levels of *RB1* and *CCND1* gene was also correlated with expression level of proliferation marker, *Ki-67*. Significant correlation was observed which is according to earlier findings that genetic aberrations and expression deregulation of cell cycle pathway genes results in uncontrolled cell cycle process and enhanced proliferation.

In conclusion, our study demonstrates a significant association between cell cycle pathway genes, polymorphisms and brain tumors in Pakistani population. Our study also confirms that the deregulated cell cycle pathway gene is linked with increased brain tumorigenesis, at least in Pakistani population. As the matter of fact, tumorigenesis and deregulated cell cycle genes are the major challenging items in neoplastic initiation and progression. Additional studies such as, a large-scale studies adjusting for a wide range of factors, [7,10] functional alteration studies as single cell level will be recommended to validate these findings and to fully explore the molecular mechanisms that contribute to the deregulation of cell cycle pathway genes in brain tumorigenesis.

## Abbreviations

CI: confidence interval
DEPC: diethyl pyrocarbonate
GBM: glioblastoma multiforme
IDT: integrated DNA technologies
IHC: immunohistochemical analysis
LD: linkage disequilibrium
OR: odds ratio
PIMS: Pakistan Institute of Medical Sciences
SNP: single nucleotide polymorphism
